# Consensus-based recommendations for optimizing perioperative nutritional support and muscle health in major surgery in India

**DOI:** 10.3389/fnut.2025.1538161

**Published:** 2025-06-05

**Authors:** Adarsh Chaudhary, B. Ravinder Reddy, G. V. Rao, Subhash Raveendran, Sanjoy Mandal, Patta Radhakrishna, Puneet Dhar, Arvind Krishnamurthy, Nilesh Doctor, Irfan Shaikh, Ankita Deora

**Affiliations:** ^1^Department of GI Surgery, GI Oncology and Bariatric Surgery, Medanta Hospital, Gurugram, India; ^2^Department of Surgical Gastroenterology and General Surgery, Care Hospitals, Hyderabad, India; ^3^Department of Surgical Gastroenterology and Minimally Invasive Surgery, Asian Institute of Gastroenterology (AIG), Hyderabad, India; ^4^Department of Surgical Gastroenterology and Hepato-Biliary Surgeon, SK Hospitals, Trivandrum, India; ^5^Department of Gastrointestinal Surgery, Manipal Hospitals, Kolkata, India; ^6^Department of Surgical Gastroenterology & Minimal Access Surgery, MGM-Malar Hospital, Chennai, India; ^7^Department of Gastrointestinal Surgery, Amrita Hospital, Faridabad, India; ^8^Department of Surgical Oncology, Cancer Institute (WIA), Chennai, India; ^9^Department of Gastrointestinal Surgery and Hepatopancreatobiliary Surgery, Jaslok Hospital and Research Center, Mumbai, India; ^10^Department of Medical Affairs and Research, Abbott Nutrition International, Mumbai, India

**Keywords:** nutrition assessment, nutritional status, sarcopenia, malnutrition, postoperative complications, nutritional support, surgical procedures

## Abstract

**Background:**

Malnutrition and sarcopenia significantly affect outcomes in patients undergoing major surgery, leading to increased postoperative complications, prolonged recovery, and higher healthcare costs. Adequate perioperative nutritional support and muscle health optimization are crucial to improving these outcomes. This study aims to provide consensus-based recommendations for integrating perioperative nutritional practices along with the standard of care in patients undergoing major surgery.

**Methods:**

A modified Delphi process was employed with a panel of experts, and recommendations were made based on a comprehensive review of current evidence.

**Results:**

The expert panel reached a high level of agreement on the importance of early nutritional screening, including muscle health evaluation and use of oral nutritional supplements, multimodal prehabilitation (including exercise and nutritional optimization), and targeted postoperative interventions to enhance recovery, maintain muscle health, and reduce complications.

**Conclusion:**

The consensus recommendations provide a clear framework for integrating effective nutritional practices into routine surgical care. These strategies have the potential to significantly improve patient outcomes and reduce healthcare costs.

## 1 Introduction

The importance of nutritional support for surgical patients is increasingly being recognized. Malnutrition is a prevalent issue that significantly impacts surgical outcomes, contributing to increased mortality, postoperative complications, and extended hospital stays (1, 2). The risk of malnutrition in general surgery patients typically ranges between 20 and 50%. For those undergoing major hepatobiliary, pancreatic, or bowel surgeries, over 80% may either be at risk of malnutrition or already malnourished (3). It has been reported that the prevalence of undernutrition in surgical patients can be as high as 3 in 5 in low- and middle-income countries (4). An assessment of preoperative cancer patients in a tertiary hospital from South India revealed that 44% were classified as malnourished (5). Patients with underlying conditions such as cancer, chronic organ failure, or inflammatory bowel disease are particularly prone to malnutrition (6). Additionally, the surgery itself can exacerbate nutritional deficits due to its catabolic effects. Like any form of trauma, surgery triggers the release of stress hormones (e.g., cortisol, catecholamines, glucagon) and inflammatory cytokines (e.g., tumor necrosis factor-alpha, interleukins 1 and 6) (7). This inflammatory response, along with increased metabolic stress, can impair immune function, lead to loss of muscle mass, and further worsen malnutrition (6). It is thus crucial to address malnutrition as a modifiable preoperative risk factor (8). Sarcopenia, a condition characterized by the progressive decline in skeletal muscle mass and strength, is a critical concern, further complicating recovery and increasing the risk of adverse outcomes (9, 10). The prevalence of sarcopenia in patients undergoing gastrectomy surgery has been reported to range between 12.5 and 57.7% (11). In addition, surgery-related muscle loss has been observed in as many as 52% of patients (12).

Evidence suggests that sarcopenia is not only associated with aging but also with malnutrition, chronic inflammation, and inadequate physical activity, all of which are exacerbated by the surgical stress response (13, 14). Given the complex interplay between nutrition, muscle health, and surgical outcomes, there is a pressing need for evidence-based protocols to optimize perioperative nutritional practices. Current research underscores the potential of multimodal prehabilitation, including exercise, nutritional optimization, and psychological support, in enhancing surgical recovery and reducing complications (15, 16). Moreover, postoperative nutritional interventions, particularly the use of oral nutritional supplements (ONS), have been shown to improve clinical outcomes and enhance the overall quality of life in patients at nutritional risk (17). Evidence in head and neck cancer patients undergoing chemoradiotherapy also underscores the critical role of nutritional support (18). These findings highlight the broader implications of nutritional interventions across major surgeries.

Despite the advances, significant challenges remain in the implementation of nutritional interventions in clinical practice. There is a need for effective educational programs that would allow the translation of scientific knowledge on nutritional aspects into clinical practice (19). This manuscript presents consensus-based recommendations developed through a modified Delphi process involving a panel of surgical experts. The aim is to provide a clear, actionable guide for optimizing perioperative nutritional support and muscle health in major surgical patients.

## 2 Materials and methods

**FIGURE 1:**
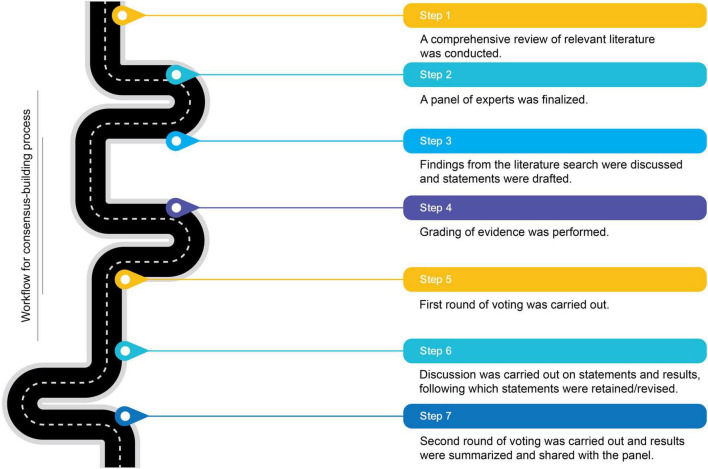
Workflow for the consensus-building process.

The primary aim of this initiative was to gather expert insights and recommendations on the clinical importance of muscle health and perioperative nutritional practices in major surgical patients, considering emerging evidence. This study employed a modified Delphi-based approach to harness collective expertise through iterative feedback. The process involved two rounds of surveys, with discussions to evaluate and revise or retain responses and statements for the second round. This study does not require IRB review as it does not involve research on human subjects. Figure 1 provides an overview of the workflow undertaken to achieve consensus.

### 2.1 Formation of an expert panel

A panel of experts was formed to leverage their experiences and insights on the topic area to ensure the relevance of the consensus-based recommendations. The panel comprised nine members, including a moderator, who were clinicians with considerable experience in their respective surgical specialties. The group consisted of experts from different domains of surgery like oncosurgery, gastrointestinal surgery, and hepatobiliary surgery. This panel participated in every step of the consensus development process.

A comprehensive literature search and review were conducted to gather up-to-date scientific evidence. The identified research articles were graded using the Oxford level of evidence (20). Based on this review, four broader topic areas were identified, and consensus statements were drafted across these areas, which underwent several iterations for refinements based on expert feedback.

### 2.2 Administering the questionnaire, gathering responses, and aggregating results

Responses were recorded via a virtual platform as ratings on a 5-point Likert scale ranging from “strongly agree” to “strongly disagree.” Results were aggregated based on predefined criteria for consensus levels:

1. High consensus: ≥ 80% agreement (agree/strongly agree or disagree/strongly disagree).
2. Moderate consensus: 60–79% agreement.
3. Low consensus: <60% agreement.

This stratification identified areas for discussion and further revision of statements for a second round of surveys.

### 2.3 Consensus development process

The consensus development process followed a modified Delphi-based method involving two iterations. After the first round of surveys, an in-person meeting was convened to facilitate discussion on topic areas and statements needing revision. Diverging views were thoroughly examined, leading to either adjustments to the statements or retention of the original perspectives. At the end of the discussion, a final round of opinions was gathered to ensure all differing views were addressed before finalizing the recommendations for the second round of voting. Following the discussions, participants were allowed to either update their original responses or keep them unchanged in a second round of online surveys.

## 3 Results

### 3.1 Consensus statements

The initial round of the Delphi process included 31 statements (out of which two were open-ended, requiring respondents to enter text-based responses). It was divided into four sections:

1. Nutrition status of surgical patients: Four statements (one open-ended)
2. The etiopathogenesis of sarcopenia: Five statements
3. Nutrition assessment: When, which tool, and why—six questions (one open-ended)
4. Benefits of nutrition intervention
  a. Prehabilitation in surgical patients: Five questions
  b. Postoperative nutrition intervention for recovery: Five questions
  c. Types of nutrition recommendations and clinical evidence: Three questions
  d. Role of certain nutrients in postoperative recovery: Three questions

The finalized consensus statements after two survey rounds are listed in Tables 1–7.

**TABLE 1:** Consensus statements: nutrition status in surgical patients.

Sl. no.	Statements in the questionnaire	Level of evidence	Level of consensus
1	Sarcopenia is associated with an increased risk of mortality, complications, and low survival rates in surgical patients.	2a, 3a	High
2	Approximately 60% of elderly patients with preoperative sarcopenia are at risk of malnutrition.	1b	High
3	Malnutrition in surgical patients is linked to an increased risk of postoperative complications.	3a, 2b	High

**TABLE 2:** Consensus statements: etiopathogenesis of sarcopenia.

Sl. no.	Statements in the questionnaire	Level of evidence	Level of consensus
4	Patients with insufficient physical activity, along with insufficient nutritional intake, are more likely to develop surgery-related muscle loss.	1b, 2b	High
5	The clinical features of sarcopenia include low L3 SMI, low handgrip strength, and reduced gait speed.	2b	High
6	Sarcopenia is associated with low albumin levels, a higher NRS 2002 score, and a higher prevalence of diabetes. However, the association between low albumin levels and diabetes with sarcopenia may not be directly causal.	2b	Moderate
7	Several Indian patients undergoing major surgery are at a high risk of malnutrition (including caloric and protein deficits) upon admission due to several factors such as dietary habits, socioeconomic conditions, preexisting health issues, and limited access to comprehensive preoperative nutritional assessment and intervention.	2b 2c	High
8	Elevated levels of inflammatory cytokines, such as CRP, IL-6, and TNFα, have been associated with a gradual decline in muscle strength and/or mass with time.	2a	High

CRP: C-reactive protein; IL-6: Interleukin-6; NRS: Nutrition risk screening; SMI: Skeletal muscle index; TNFα: Tumor necrosis factor-α.

**TABLE 3:** Consensus statements: nutritional assessment.

Sl. no.	Statements in the questionnaire	Level of evidence	Level of consensus
9	The SGA can be used as one of the nutrition assessment tools for surgical patients.	2a	High
10	Some of the most common variables used to diagnose malnutrition are albumin, mid-arm muscle circumference, BMI, cholinesterase, hemoglobin, neutrophil-to-lymphocyte ratio, and the total number of lymphocytes.	2a	High
11	Low skeletal muscle mass is a significant risk factor for postoperative complications in patients with cancer.	2a	High
12	CT is a commonly used technique for assessing muscle mass; however, its use exclusively for this purpose is optional.	2a	High
13	Nutritional assessment is important in selecting at-risk surgical patients who need intervention.	2a	High

BMI, Body mass index; CT, Computed tomography; SGA, Subjective global assessment.

**TABLE 4:** Consensus statements: prehabilitation in surgical patients.

Sl. no.	Statements in the questionnaire	Level of evidence	Level of consensus
14	Prehabilitation improves preoperative functional exercise capacity, improves muscle strength, and is associated with fewer postop complications in patients with sarcopenia.	1b, 1a	High
15	Preoperative nutrition support could help reduce complications in patients undergoing cancer surgery or abdominal surgery.	1b, 1a	High
16	Preoperative nutrition support can be provided through HEHP drinks, immunonutrition, enteral nutrition, and parenteral nutrition.	1b	High
17	Preoperative carbohydrate loading is more beneficial than keeping patients NBM.	1a	High
18	Preoperative nutritional supplementation significantly improves postoperative serum albumin levels in Indian patients undergoing major surgeries. However, there is no evidence to support the use of albumin infusions parenterally.	1b 1b	High

HEHP, High energy high protein; NBM, Nil by mouth.

**TABLE 5:** Consensus statements: Postoperative nutrition intervention for recovery.

Sl. no.	Statements in the questionnaire	Level of evidence	Level of consensus
19	Early oral feeding is preferred for surgical patients.	1b	High
20	Postoperative nutrition intervention via ONS is known to reduce the incidence of sarcopenia after 3 months of intervention.	2b	High
21	A combination of EN and PN has been found to reduce protein deficits in malnourished patients undergoing major surgery.	2b	High
22	The addition of immunonutrients to postoperative diets does not reduce postoperative complications in patients with cancer.	1b, 1a	Low
23	Peptide-based nutrition can be used for priming or in cases of suspected gastrointestinal intolerance following major surgeries.	2b, 1a	High

EN, Enteral nutrition; ONS, Oral nutritional supplements; PN, Parenteral nutrition.

**TABLE 6:** Types of nutrition recommendations and clinical evidence.

Sl. no.	Statements in the questionnaire	Level of evidence	Level of consensus
24	Nutritional interventions are predominantly subdivided into standard ONS, oral immunonutrition supplements, weight loss interventions, oral prebiotics and probiotics, dietary optimization of comorbidities, and others (generally including administration of nutritional supplements such as EPA, HMB, antioxidants, etc.).	1a	High
25	To prevent muscle wasting and loss of lean mass after obesity surgery, protein can be added to the diet by directly calculating the required amount based on adjusted body weight, with a minimum of 1–1.5 g of high biological value protein per kg of weight per day.	3a	High
26	3 g/day of HMB combined with exercise may benefit adults with sarcopenia by enhancing or maintaining muscle mass, strength, and function despite muscle loss.	1b	High

EPA, Eicosapentaenoic acid; HMB, Beta-hydroxy beta-methylbutyrate.

**TABLE 7:** Consensus statements: role of certain nutrients in postoperative recovery.

Sl. no.	Statements in the questionnaire	Level of evidence	Level of consensus
27	A balanced oral nutritional supplement with adequate calories, high-quality protein, vitamins, and minerals helps improve BMI and SMI after surgery.	2b	High
28	Preoperative nutrition supplementation (containing L-arginine, L-glutamine, and HMB) reduces the CPK-MB, troponin, and SOFA scores; the time of food intake; and the duration of stay in hospitalized patients.	1b	High
29	Increased protein intake, combined with physical activity, is associated with a reduced risk of developing surgery-related muscle loss.	2b	High

BMI, Body mass index; CPK-MB, Creatine phosphokinase-myocardial band; HMB, Beta-hydroxy beta-methylbutyrate; SMI, Skeletal muscle index; SOFA, Sequential organ failure assessment.

## 4 Discussion

### 4.1 Nutrition status in surgical patients

Sarcopenia is characterized by a progressive and global decline in skeletal muscle mass and strength. It is typically associated with aging, immobility, or the status of illness (10). Age-associated decreases in muscle mass can be 3–8% per decade after the age of 30 years, with the decline rate being even higher after 60 years (21). Although chronological age has been used as a predictor of postoperative outcomes, sarcopenia has been proposed as a more accurate risk predictor (9, 10). Evidence from a systematic review and meta-analysis of 294 studies suggests that sarcopenia is a significant predictor of poor outcomes after surgery, with higher mortality, complications, and lower survival rates, regardless of procedural type (9). Sarcopenia also influences the length of hospital stay and the rate of home discharge (9, 10). Given such influence on outcomes, with a rise in median age globally, preemptive identification of vulnerable patients and anticipating potential postoperative complications are becoming increasingly important (9). Malnutrition has been reported to be an independent predictor of sarcopenia. A study evaluating screening tools for sarcopenia among cancer patients aged > 60 years scheduled for surgery reported that about 60% of elderly patients with sarcopenia were at risk for malnutrition (22). In addition to increased length of stay and risk of mortality, malnutrition among surgical patients can lead to an increased risk of postoperative complications, such as surgical site infection, delayed wound healing, bleeding, and delayed ambulation (1, 2). The consensus statements for this section are outlined in Table 1.

### 4.2 Etiopathogenesis of sarcopenia

Insufficient preoperative physical activity and inadequate nutritional intake are independent risk factors for surgery-related muscle loss, exacerbated by surgical trauma, which triggers a catabolic response leading to accelerated muscle loss after surgery (13). Skeletal muscles play a critical role in systemic metabolism and energy homeostasis. Patients with sarcopenia often exhibit a low skeletal muscle index (SMI), reduced handgrip strength, and slow gait speed. This condition is associated with older age, lower albumin levels, lower body mass index (BMI), reduced hemoglobin levels, higher Nutritional Risk Screening (NRS) scores, and a higher prevalence of diabetes (23). However, the direct relationship between sarcopenia and blood albumin levels remains unconfirmed (24). Major gastrointestinal (GI) surgery patients, especially those with malignancies, are at significant risk of malnutrition, necessitating perioperative nutritional support to minimize complications. A substantial proportion of Asian GI surgery patients experience postoperative nutritional deficits due to preoperative malnutrition or the risk thereof, highlighting the urgent need for improved nutritional support and education (19). Additionally, chronic inflammation is closely linked with sarcopenia and its components, including skeletal muscle strength and muscle mass, with higher levels of circulating inflammatory markers significantly associated with lower muscle strength and mass, indicating the critical role of systemic inflammation in the pathogenesis of sarcopenia (14). These insights underscore the importance of comprehensive preoperative assessments and targeted nutritional interventions to enhance surgical outcomes and overall patient health. The consensus statements for this section are outlined in Table 2.

### 4.3 Nutritional assessment: when, which tool, and why

Perioperative malnutrition is prevalent and linked with higher mortality rates, increased complications, and elevated healthcare costs. Patients undergoing surgery for cancer or GI diseases are especially vulnerable (8). As a modifiable preoperative risk factor, nutritional assessment is important to identify at-risk individuals and ideally position interventions that can enhance outcomes. There was a high level of agreement among the experts regarding the use of Subjective Global Assessment (SGA) for the nutritional assessment of surgical patients. SGA is the most commonly used tool that is considered reliable and has high reproducibility (25, 26). SGA is a simple, safe, and inexpensive tool validated for diagnosing malnutrition in diverse patient populations. Given its ease of use, clinical applicability, and practical considerations specific to Indian settings, the panel recommended its use. While several validated tools exist for assessing nutritional risk in surgical patients, such as the Nutritional Risk Screening (NRS 2002), Malnutrition Universal Screening Tool (MUST), and Mini Nutritional Assessment (MNA), the SGA was preferred by the expert panel. SGA allows for immediate classification of nutritional status without requiring advanced measurements or calculations, which is particularly valuable in resource-limited settings or during preoperative assessments when rapid decision-making is essential. In this context, the Global Leadership Initiative on Malnutrition (GLIM) criteria are a comprehensive tool designed to build global consensus around core diagnostic criteria (27). However, the panel discussion highlighted that GLIM criteria require parameters that might not be routinely assessed in surgical patients in Indian settings. It was suggested that some of the alternative nutrition markers that can be used include albumin, mid-arm muscle circumference, BMI, cholinesterase, hemoglobin, neutrophil-to-lymphocyte ratio, and the total number of lymphocytes. These parameters have been reported to be used for identifying malnutrition in emergency general surgery settings (26). Among these, studies show that low cholinesterase levels correlate with worse surgical outcomes, especially in patients undergoing surgery, where malnutrition significantly impacts recovery (28, 29). Supported by evidence, there was agreement that low skeletal mass can be considered an independent risk factor for postoperative complications in patients with cancer (30).

Computed tomography (CT) is considered the gold standard for muscle quantification in patients with sarcopenia (9). Studies have reported its widespread use for assessing muscle mass (30). The expert panel shared that, although in their experience, CT is commonly used, the associated radiation exposure could be a disadvantage. Therefore, recommending CT as a screening tool for patients who do not need it for any other reasons may not be optimal (31). The discussion also highlighted that while bioelectrical impedance analysis (BIA) and dual-energy X-ray absorptiometry (DEXA) are valuable tools for assessing muscle mass, incorporating CT in practice remains feasible due to its established clinical use and accessibility. CT scans are routinely performed preoperatively. This makes it a readily available opportunistic method to assess skeletal muscle mass without requiring additional procedures. Also, India-specific cut-offs for muscle area and density as markers of sarcopenia have been proposed (32). Further research should be encouraged from different regions of India to optimize the Indian standards. Additionally, functional measures like handgrip strength and the 6-min walk test (6MWT) can provide valuable insights into muscle function and surgical recovery. The consensus panel acknowledged the growing role of these alternative modalities, and the discussion supported their integration into perioperative assessment protocols. The consensus statements for this section are outlined in Table 3.

### 4.4 Benefits of nutritional intervention

#### 4.4.1 Prehabilitation in surgical patients

Prehabilitation, defined as the process of enhancing an individual’s functional capacity to withstand a stressful event better, has become increasingly recognized for its potential to improve surgical outcomes. Despite this recognition, there are missed opportunities to optimize modifiable risk factors such as preexisting comorbidities, fitness levels, nutritional status, psychological wellbeing, and the adverse effects of neoadjuvant therapies (15). Multimodal surgical prehabilitation, which includes interventions such as exercise, nutrition optimization, smoking and alcohol cessation, and psychological stress reduction, is gaining traction worldwide (33). Implementation of prehabilitation has the potential to significantly benefit patients (especially those with sarcopenia) by improving muscle strength and exercise capacity, in addition to reducing postoperative complications and improving recovery times (16, 34, 35). Along these lines, there was also agreement that preoperative nutrition support for patients undergoing cancer or abdominal surgery can reduce complications. Such benefits have been demonstrated even among non-malnourished patients with cancer undergoing surgery (36). Implementation of preoperative nutritional supplementation has been shown to improve clinical outcomes, including postoperative serum albumin (37). There was agreement that nutrition support can be provided via high-energy, high-protein drinks, immunonutrition, enteral nutrition (EN), and parenteral nutrition (PN) (38, 39). Preoperative carbohydrate-rich drinks may reduce the negative effects of surgical stress and fasting, such as insulin resistance and delayed recovery. A systematic review of 17 randomized controlled trials involving 1,445 patients found that these drinks significantly improved insulin resistance and indices of patient comfort after surgery without adverse events such as aspiration (40). The expert panel emphasized that multimodal prehabilitation remains beneficial, particularly for high-risk patients. While there is no uniform consensus among Indian surgeons, the panel supported a personalized approach based on patient risk stratification rather than a universal prehabilitation protocol. Further India-specific research is needed to refine prehabilitation strategies and determine the most effective components for improving perioperative outcomes. The consensus statements for this section are outlined in Table 4.

#### 4.4.2 Postoperative nutrition intervention for recovery

Early feeding is considered one of the key components of facilitating early recovery while improving patient outcomes and experiences (41). Results from a systematic review and meta-analysis of 34 randomized controlled trials (RCTs) indicate that early oral feeding after GI surgery may lead to faster intestinal recovery, shorter postoperative stays, and fewer complications (42). The expert panel was in agreement regarding early oral feeding, acknowledging the role of postoperative ONS in reducing the incidence of sarcopenia. Postdischarge ONS, combined with dietary advice, has been shown to significantly improve nutritional outcomes, skeletal muscle maintenance, and chemotherapy tolerance in patients with gastric cancer at nutritional risk compared with dietary advice alone. It was found that patients receiving ONS had less weight loss, higher BMI and SMI, a lower incidence of sarcopenia, fewer chemotherapy modifications, and improved quality of life (in terms of fatigue and appetite loss) (17). Patients receiving a combination of EN and PN have been reported to experience the lowest caloric and protein deficits (19). Such findings suggest that supplementary parenteral nutrition (PN) may provide a more adequate nutritional treatment for individuals who tolerate enteral nutrition (EN) administration. There was a low level of agreement on the statement that the addition of immunonutrients to postoperative diets does not reduce postoperative complications in patients with cancer. Such a lack of agreement may be a result of the debatable nature of the advantages of immunonutrition for patients requiring surgery, especially those with GI neoplasms (43, 44). Although early initiation of EN is important, some patients may experience gut intolerance. There was agreement that the use of peptide-based nutrition in such patients may be beneficial. It has been shown that dipeptide- and tripeptide-based EN can be more efficacious and better tolerated than whole protein-based formulas (45). The expert panel acknowledged that while EN is generally preferred, there are postoperative situations where EN is not feasible due to factors like gut intolerance or surgical complications. In such cases, PN can serve as a critical alternative. The discussion supported the idea that PN should be considered when enteral feeding is not possible, ensuring that nutritional needs are met to support recovery and minimize the risk of further muscle loss and complications. The consensus statements for this section are outlined in Table 5.

#### 4.4.3 Types of nutrition recommendations and clinical evidence

To improve the nutritional status of surgical patients, a range of evidence-based interventions can be applied depending on the patient’s risk profile and type of surgery. According to the ESPEN guidelines, assessment of nutritional status is recommended before and after major surgery. Nutritional interventions can be predominantly subdivided into standard ONS, oral immunonutrition supplements, weight loss interventions, oral prebiotics and probiotics, nutritional optimization, and others [generally including administration of nutritional supplements such as eicosapentaenoic acid (EPA), beta-hydroxy beta-methylbutyrate (HMB), antioxidants, etc.] (46). Standard ONS are particularly indicated for patients unable to meet nutritional needs through regular intake (47). If the energy and nutrient requirements cannot be met by oral or enteral intake alone (<50% of caloric requirement) for more than 7 days, ESPEN recommends initiating a combination of enteral and parenteral nutrition. For immunonutrition, supplements enriched with arginine, omega-3 fatty acids, and nucleotides are particularly useful in high-risk patients, such as those undergoing gastrointestinal or oncologic surgery (48). ESPEN guidelines recommend peri- or atleast post-operative administration of specific formula enriched with such ingredients for malnourished patients undergoing surgery (47). In agreement with the Andalusian Group for Nutrition Reflection and Investigation (GARIN), the expert panel also recommended that protein be added to the diet to prevent muscle wasting and lean muscle loss after obesity surgery. The suggested calculation was based on adjusted body weight, with a minimum of 1–1.5 g of high biological value protein per kg of weight per day (49). HMB, an essential branched-chain amino acid, has been shown to improve muscle mass and function. However, its role in sarcopenia remains inconclusive. The panel reviewed existing evidence and recommended that there may be an advantage in combining HMB (3–6 g/d) with resistance exercise training to improve the effect later (50). Prebiotic (oligosaccharides such as inulin and galacto-oligosaccharides) and probiotic supplementation are also becoming more relevant. Preoperative plus postoperative synbiotics are more effective than only postoperative synbiotics or placebo, reducing the incidence of infections, with less hospital stay and length of antibiotic usage (51, 52). Additional micronutrients and antioxidant optimization are also essential. Vitamin D, selenium, zinc, and vitamin C support immune function, reduce oxidative stress, and promote tissue healing (53). EPA also exhibits anti-inflammatory effects and may help preserve lean body mass during cancer treatment (54). Collectively, these interventions, when initiated early and delivered through a multidisciplinary approach, play a critical role in optimizing surgical recovery and reducing perioperative risk. The consensus statements for this section are outlined in Table 6.

#### 4.4.4 Role of certain nutrients in postoperative recovery

Studies have indicated that malnutrition is consistently associated with poor outcomes, especially after discharge, among patients undergoing major surgery. This leads to high incidence of morbidity and mortality, low chemotherapy tolerance, and short survival time (17). To overcome this challenge, the panel recommended postoperative balanced oral nutritional supplementation with adequate calories, high-quality protein, vitamins, and minerals to help improve the BMI and SMI. Such supplementation has been reported to improve nutritional outcomes, skeletal muscle maintenance, chemotherapy tolerance, and some quality-of-life variables among patients with gastric cancer (17). Nutrition deficiencies may cause inflammation by altering the function of the defense system and depleting the antioxidative nutrient reserve. Depletion of micronutrients and macronutrients can make patients susceptible to sarcopenia, cachexia, surgical trauma, and infection. In this regard, the role of supplementation with L-glutamine, HMB, and L-arginine (Gln/HMB/Arg) has been evaluated in patients undergoing heart surgery. The results highlighted that perioperative supplementation with such a combination reduces creatine phosphokinase-myocardial band (CPK-MB), troponin, and sequential organ failure assessment (SOFA) scores. It also positively influenced the time of food intake and length of stay (55). Based on such findings, the panel agreed on the perioperative use of Gln/HMB/Arg supplementation. In postoperative patients, surgical trauma plays a critical role in the catabolic response, resulting in muscle loss. An assessment of the combination of insufficient physical activity and nutritional intake reported higher rates of surgery-related muscle loss (13). There was a high level of agreement regarding increased protein intake combined with physical activity to reduce the risk of developing surgery-related muscle loss. The consensus statements for this section are outlined in Table 7.

### 4.5 Suggested algorithm for perioperative nutrition therapy

**FIGURE 2:**
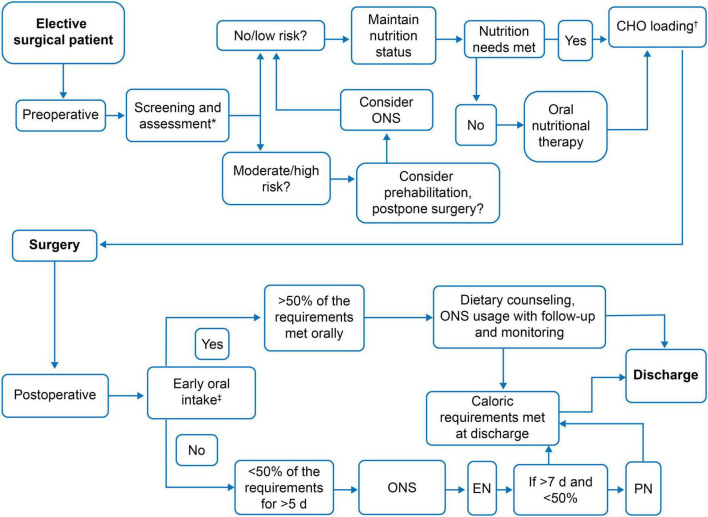
Suggested algorithm for perioperative nutrition therapy. *Individual preference supported by evidence. ^†^Caution is required in people with diabetes. ^‡^As early as possible. CHO, Carbohydrate-rich; EN, Enteral nutrition; ONS, Oral nutritional supplement; PN, Parenteral nutrition.

The discussion revolved around the need for a simplified algorithm that can provide a preliminary scaffold for developing and implementing nutritional practices. The panel reviewed the algorithm provided by the existing European Society for Clinical Nutrition and Metabolism (ESPEN) practical guidelines, simplifying it for ease of adoption in the Indian setting (47). The recommendation to implement selective prehabilitation rather than a universal approach was based on practical considerations in the Indian healthcare setting. Resource constraints, patient compliance challenges, and variations in surgical risk profiles necessitate a targeted approach to optimize feasibility and impact. Figure 2 outlines the overall framework for perioperative nutrition therapy.

### 4.6 Insights on barriers and challenges to implementing nutritional interventions in India

The discussion also highlighted several barriers and challenges to integrating nutrition into surgical practice, along with potential solutions (Table 8). Key issues included the lack of a dedicated team and the absence of standard operating procedures to monitor and audit nutritional interventions. There is also a significant disconnect between the perspectives of caregivers, compounded by the limited time allocated for prehabilitation. Additionally, the prevalent vegetarian diet in India poses challenges to ensuring adequate micronutrient intake. The need for formulations tailored to Indian patients was pointed out. The panel emphasized the importance of sensitizing newer physicians to the nuances of nutrition, particularly the role of nutrition in preventing sarcopenia. Financial constraints and the fact that insurance does not cover nutritional support were identified as major barriers. The discussion also stressed the need for nutritional support across all types of surgeries and incorporating comprehensive nutrition education into the medical curriculum. The inclusion of nutrition topics in medical conferences was recommended to raise awareness and promote best practices. Finally, the importance of disseminating knowledge in a context-specific manner to facilitate adoption was underscored.

**TABLE 8:** Summary of outcomes from the discussion: practice points.

Preoperative phase
• Nutritional screening and assessment: Implement early nutritional screening using validated tools such as the Subjective Global Assessment (SGA) to identify patients at risk of malnutrition and sarcopenia.
• The clinical features of sarcopenia include low L3 skeletal muscle index (SMI), low handgrip strength, and reduced gait speed. Computed tomography (CT) scans can be used for muscle mass evaluation, particularly in patients with a high risk of sarcopenia.
• Multimodal prehabilitation: Prehabilitation programs should include exercise, nutritional optimization, and psychological support to improve physical function and muscle strength, especially in patients with sarcopenia. This can reduce postoperative complications and enhance recovery.
• Preoperative nutritional support: Patients undergoing cancer or abdominal surgery should receive nutritional support, including high-energy, high-protein oral nutritional supplements (ONS), and immunonutrition. Preoperative carbohydrate loading is recommended over fasting to enhance postoperative recovery.
**Intraoperative phase**
• Combined enteral and parenteral nutrition: For patients with significant caloric and protein deficits, consider combining enteral nutrition (EN) and parenteral nutrition (PN) to meet nutritional needs during the perioperative period. This approach helps prevent protein-energy malnutrition.
**Postoperative phase**
• Early oral feeding: Initiate early oral feeding as soon as possible after surgery to facilitate faster recovery and reduce complications, such as delayed intestinal function. This is especially important for gastrointestinal surgery patients.
• Postoperative oral nutritional supplementation: Oral nutritional supplements (ONS) should be continued postoperatively for at least 3 months to reduce the incidence of sarcopenia and improve body mass index (BMI) and skeletal muscle index (SMI). This approach also enhances chemotherapy tolerance and quality of life.
• Targeted nutritional interventions: Supplementation with specific nutrients, such as L-arginine, L-glutamine, and beta-hydroxy-beta-methylbutyrate (HMB, 3–6 g/d), has been shown to reduce inflammation, improve immune function, and promote muscle preservation in postoperative patients.
• Protein intake and physical activity: Postoperative patients should be encouraged to increase their protein intake (1–1.5 g per kg of body weight per day) and engage in physical activity to reduce surgery-related muscle loss and improve functional outcomes.
• Management of gastrointestinal intolerance: In patients with gastrointestinal intolerance, peptide-based enteral formulas (dipeptide- and tripeptide-based) can be used to enhance tolerance and improve nutritional outcomes.
• Monitor and adjust nutritional plans: Regularly monitor nutritional intake and outcomes (e.g., weight, muscle mass, albumin levels) postoperatively to adjust nutritional interventions and ensure optimal recovery.

## 5 Conclusion

This manuscript provides consensus-based recommendations for optimizing perioperative nutritional support and muscle health in patients undergoing major surgery. The expert panel emphasizes the critical role of addressing malnutrition and sarcopenia to improve surgical outcomes. Key recommendations include the use of ONS, multimodal prehabilitation, and targeted postoperative interventions to enhance recovery, maintain muscle mass, and reduce complications. Despite clear benefits, challenges such as regional dietary habits and a lack of standardized protocols hinder implementation. These recommendations offer actionable strategies to integrate effective nutritional practices into routine care, ultimately improving patient outcomes and reducing healthcare costs. Continued efforts are necessary to promote the adoption of these best practices. Furthermore, focused initiatives are needed to optimize nutritional strategies tailored to specific conditions, such as chronic kidney disease or sarcopenic obesity. Extending the efforts to condition-specific perioperative nutrition regimens will allow patient-centered care and improve clinical outcomes.

## Data Availability

The original contributions presented in this study are included in this article/supplementary material, further inquiries can be directed to the corresponding author.
